# Predator and prey functional traits: understanding the adaptive machinery driving predator–prey interactions

**DOI:** 10.12688/f1000research.11813.1

**Published:** 2017-09-27

**Authors:** Oswald Schmitz

**Affiliations:** 1 School of Forestry and Environmental Studies, Yale University, 370 Prospect Street, New Haven, CT, 06515, USA

**Keywords:** Predator, Prey, Functional Traits, Evolution, Ecology, Adaptations

## Abstract

Predator–prey relationships are a central component of community dynamics. Classic approaches have tried to understand and predict these relationships in terms of consumptive interactions between predator and prey species, but characterizing the interaction this way is insufficient to predict the complexity and context dependency inherent in predator–prey relationships. Recent approaches have begun to explore predator–prey relationships in terms of an evolutionary-ecological game in which predator and prey adapt to each other through reciprocal interactions involving context-dependent expression of functional traits that influence their biomechanics. Functional traits are defined as any morphological, behavioral, or physiological trait of an organism associated with a biotic interaction. Such traits include predator and prey body size, predator and prey personality, predator hunting mode, prey mobility, prey anti-predator behavior, and prey physiological stress. Here, I discuss recent advances in this functional trait approach. Evidence shows that the nature and strength of many interactions are dependent upon the relative magnitude of predator and prey functional traits. Moreover, trait responses can be triggered by non-consumptive predator–prey interactions elicited by responses of prey to risk of predation. These interactions in turn can have dynamic feedbacks that can change the context of the predator–prey interaction, causing predator and prey to adapt their traits—through phenotypically plastic or rapid evolutionary responses—and the nature of their interaction. Research shows that examining predator–prey interactions through the lens of an adaptive evolutionary-ecological game offers a foundation to explain variety in the nature and strength of predator–prey interactions observed in different ecological contexts.

## Introduction

Most animal species are engaged in a predator–prey relationship by consuming prey or falling victim to predators or both. Predator–prey relationships are therefore a mainstay of community ecology. Thus, characterizing these relationships functionally offers a way to understand the complexity that is inherent in predator–prey communities ^1– 5^.

**Figure 1.**
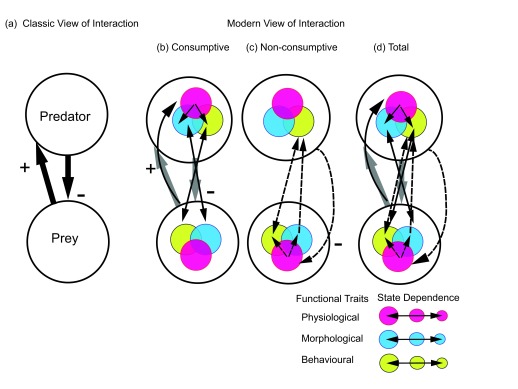
Depiction of classic and modern views of a predator–prey interaction. A predator–prey interaction is represented as a module where consumptive effects are depicted by solid arrows and non-consumptive effects by dashed arrows. ( **a**) In the classic, generic view, predators have a negative consumptive effect on prey, and prey provide a positive nutritional benefit to predators. ( **b**– **d**) A modern view considers greater complexity due to interplay between predator and prey functional (physiological, morphological, and behavioral) traits. The predator–prey interaction is then decomposed into consumptive and non-consumptive effects. ( **b**) The success of the predator consumptive effect on prey is contingent on the alignment (double-headed arrows) between predator morphology (for example, gape width) and prey morphology (body size) and between predator behavior (for example, hunting mode) and prey behavior (for example, escape mode). The consumption of prey feeds directly to support predator physiological needs (the nutrient balance among maintenance, growth, and reproduction). The specific example here shows that physiology then directly determines predator morphology (for example, increased size) and behavior (for example, increased aggression), although behavior could also reciprocally determine physiology and morphology. ( **c**) A non-consumptive effect could arise when a predator has a negative effect on prey by eliciting a physiological stress response. Stress in turn alters prey physiology (for example, heightened metabolism), behavior (for example, alertness and vigilance), and morphology (for example, induction of escape morphology). ( **d**) The combination of consumptive and non-consumptive interactions leads to a total predator–prey interaction that becomes an adaptive game involving changes and feedbacks between predator and prey traits. The interactions are depicted in terms of predator and prey traits of a certain magnitude (size of circles). The nature of the game, however, can vary depending on the state of predator and prey (inset) in relation to each other, as determined by the magnitude of each other’s functional traits (size of the circles). Thus, the strengths of effects may depend on how the magnitude of physiological (for example, good condition [larger] or poor condition [smaller]), morphological (for example, large bodied [larger] or small bodied [smaller]), and behavioral (for example, bold [larger] or shy [smaller]) traits of predators and prey play off against each other in the adaptive, state-dependent game.

Predator–prey relationships are classically viewed as consumptive acts between two species ^6^. This often assumes that all predators are functionally equivalent—merely capturing and consuming prey—and that all prey are passive victims. This further assumes that the nature and strength of interaction between a predator and prey species do not vary among environmental contexts. But these assumptions are inconsistent with growing evidence that much variety underlies many predator–prey relationships ^2, 7– 9^. Hence, recent analyses focus on explaining the mechanisms determining the nature of predator and prey interactions in different environmental contexts ^4, 10– 12^. In particular, explaining context dependency in the interaction between any given species means that it is insufficient to examine predator–prey relationships solely on the basis of taxonomic identity (for example, wolves and pike are predators; elk and stickleback are prey) ^4, 13– 15^. Instead, it requires considering predators and prey in terms of their functional traits ( Figure 1). A functional trait is any morphological, behavioral, or physiological trait of an organism that is associated with a biotic interaction or ecological function of interest ^4, 14^. Here, I offer a sketch of recent advances in applying a functional trait approach to better understand and predict the context-dependent nature of predator–prey interactions.

## Predator–prey functional traits

Modern conceptions view predator–prey interactions as adaptive foraging games where the success of predators is determined by their ability to capture and subdue their prey and the success of prey is determined by their ability to evade or fend off their predators ^16– 18^. Each player’s success is determined fundamentally by its functional traits relative to the other ^18^. Hence, traits that determine the ability of predators to successfully capture prey are called “foraging” or “trophic” traits, and traits that determine the ability of prey to avoid being preyed upon are called “vulnerability” traits ^5, 12, 19, 20^. Predator foraging traits include body size (mass or length), gape size, hunting or foraging mode (for example, ambush or active hunting), and feeding mode (for example, chewing or suctorial) ^5, 12, 20^. Prey vulnerability traits include body size (length and mass), body shape, defense (for example, physical or chemical protection), ability to avoid detection (crypsis or camouflage), mobility, ability to recognize and detect predators, and evasive or escape behavior (which can be elicited by physiological trait responses ^21, 22^) ^5, 12, 20^.

But whether or not predators engage with prey, or prey respond to predators in the first place, depends fundamentally on their relative body sizes (or predator gape and prey body shape). Pursuing prey that are too large or cumbersome can be harmful or energetically costly to predators, and pursuing prey that are too small may not be worth the energetic return ^23– 27^. Thus, size selectivity is the foundation for analyses of predator–prey interactions. It determines basic patterns in predator–prey relationships—who is capable of eating whom—within ecological communities ^20, 24, 27– 31^ and predation rate ^32^, but consideration of predator–prey interactions in terms of size alone is not enough. New research shows that, even when size ratios of predator and prey align, the nature of predator–prey interactions will further depend on other functional traits.

For instance, the nature of the predator–prey interaction can vary spatially owing to variation in predator hunting and feeding mode and prey mobility ^20, 33– 36^. Sit-and-wait ambush predators are more effective at capturing actively moving prey, whereas actively roaming predators are more effective at capturing sedentary prey ^33, 34^. Predator feeding mode may determine the size range of prey consumed. Size for size, aquatic sucking predators like water bugs and diving beetles can capture and consume a much larger range of prey sizes than chewing predators like dragonfly larvae and some other diving beetles can ^20^. Predator–prey interactions may be influenced by herbivore prey feeding mode, which is linked to anti-predator defense traits ^30^. Herbivores can be plant specialists or generalists and exhibit leaf chewing (grazing and browsing), sap feeding, or leaf mining feeding behavior ^37^. Specialization can be an evolved response to predators, especially in insect herbivores that are able to enlist characteristics of their host plants for defense or refuge ^37^. Lepidopteran caterpillars often sequester plant toxins that make them unpalatable or toxic to their predators ^37^, and other specialist insects become cryptic by mimicking the structural traits or coloration of their host plants ^37– 39^. Generalist herbivores select a variety of plants, making such specific defense traits ineffective. Hence, predators may have weaker effects when specialist herbivores predominate communities than when generalist herbivores are dominant ^37^. Moreover, many specialist herbivores are leaf miners and sap feeders that tend to be sedentary whereas generalists roam more widely. Hence, the strength of predator–prey interactions could be contingent on predator hunting mode ^34^.

These examples underscore the notion that predator–prey interactions functionally integrate locomotor, foraging, and defense traits, leading to a rising interest in understanding the biomechanics—the relationship between form and function—underlying these trait associations ^39, 40^. Some fish predators capture prey by using suction feeding to draw in prey ^40^, but, to be effective, predators must stealthily maneuver into close proximity without startling the prey ^40^. This requires coordinating a suite of biomechanical factors, including approach speed, acceleration, positioning, and maintaining positional stability in a three-dimensional fluid. Thus, biomechanics offers understanding of how overall body plan—the coordinated suite of functional traits—can determine success or failure in the predator–prey game. Such integrative understanding helps offer general principles about predator–prey interactions among a diversity of predator and prey species.

From a functional standpoint, the diversity of predator hunting behavior can be organized into three broad categories: ambush (sit-and-wait), ballistic interception (sit-and-pursue), or pursuit (active or coursing) ^36, 39^. Hunting modes integrate predator traits in different ways. Ambush predators lie in wait for prey. This requires being cryptic, blending into the background through posture or coloration, or hiding in physical structures like low-lying depressions or burrows or among vegetation. Their effectiveness comes from bursting forth before prey can sense and react to the attack ^39^. Their success is enhanced through heightened visual and olfactory acuity to ensure that they attack only when prey encounter is imminent ^39^. Prey may escape the attack if they have heightened sensory acuity such as the ability to detect subtle changes in airflow or waterflow as the predator begins its acceleration ^41^. Pursuit predation involves high-speed chases with rapid acceleration by the predator and rapid evasive turning by the prey ^39^. Predators must be highly reactive, constantly re-estimating prey movement directions to run them down and subdue them ^39^. An intermediate hunting mode is ballistic interception, where a predator lies in wait but burst attacks prey from farther distances than do ambush predators. Correspondingly, prey need to adjust their flight distance and be poised to flee sooner than when confronting ambush predators. Predators in turn must anticipate the movement directions of the prey in order to run them down, much like pursuit predators ^39^. All of these interactions require tight coordination between neurosensory and musculoskeletal traits as predator and prey performance dynamically changes. Consequently, predator species with similar hunting modes tend to have similar effects on prey behavior and abundance ^36^.

Changing environmental contexts that cause changes in hunting mode should accordingly lead to morphological changes related to locomotor biomechanics. Indeed, individual Aegean wall lizards live and hunt both in their ancestral sandy habitats and in agrarian habitats containing rock walls. Recent work has shown that individuals on rock walls are ambush hunters, jumping from rock to rock, but that individuals in sandy habitats engage in active running pursuit ^42^. To facilitate jumping, individuals on rock walls have longer hindlimb-to-forelimb ratios than do their sandy habitat counterparts ^42^. These hunting mode differences lead to dietary differences, where sandy habitat hunters consume more sedentary prey and rock wall hunters consume more mobile prey ^42^. Eurasian perch compete for resources within pelagic habitats and this leads to within-population divergence in habitat usage such that some population members use littoral zones ^43^. This habitat shift leads to morphological shifts, where individuals in pelagic habitats tend to have a streamline body form suited for pursuit whereas littoral individuals have deeper rounded body forms more suitable for slower maneuverability ^43^. Pelagic individuals in turn tend to have a narrower diet breadth and specialize less and thus have more stable trophic interactions than do littoral individuals who occupy a habitat that provides a higher diversity of resources ^43^.

A biomechanics perspective reveals the importance of considering functional traits in action, yet many synthetic frameworks that tend to classify species by functional traits treat those traits as fixed characteristics of species ^4^. This merely substitutes one classification scheme (taxonomic) for another (functional traits) and thus risks substituting one form of typology (species) for another (functional traits) ^4^. This limits the insights that can come from a functional trait approach.

Newer functional trait approaches help overcome typological thinking by recognizing that species comprise individuals that vary in their traits ^4, 9, 25, 27, 44, 45^. Moreover, individuals can flexibly adjust the expression of their traits to maximize survival and reproduction (fitness) ^4, 44^. Both considerations help us understand and predict context dependency. For instance, whenever a predator–prey relationship is non-linear, the mean (expected) net effect of a species may not simply reflect the species mean (expected) value of the functional trait ^27, 45^. The net effect may depend on the magnitude of trait variation and the shape of the trait distribution (for example, normal versus skewed) ^27, 45^. Individuals within a population may also adaptively adjust their trait expression to match their phenotype to the new environmental context ^44^. Ultimately, then, a functional trait approach dynamically connects evolutionary ecology with community ecology ^4, 39, 40, 44^, reinforcing the idea that a predator–prey relationship is an adaptive game ^18^.

## The evolutionary ecology of functional traits

Understanding of predator–prey interactions fundamentally changed when it was recognized that predators can exert strong non-consumptive effects on prey ^46, 47^. This realization led to the general principle that predator–prey interactions are essentially an evolutionary ecological trade-off game involving trait changes via phenotypic plasticity and adaptation via selection ^4, 44^. Early work largely explored the trade-off in terms of prey behavioral and morphological plasticity ( Figure 1) ^7, 47, 48^. Hence, prey are not unwitting victims. They behaviorally evade predators by becoming vigilant, shifting their foraging time budget, or shifting between foraging habitats and refuge habitats. Prey also induce anti-predator defense morphology, becoming cumbersome to handle by gape-limited predators, such as through the production of spines by zooplankton or through accelerated development by tadpoles to reach a predation size refuge. Prey could also become better at evading predators such as by changing the development of musculature related to swimming ^47, 48^. Predators in turn change their tactics to try to overcome prey defenses, setting up an eco-evolutionary game ^16, 18^.

More recent work shows that plastic responses of prey to perceived predation risk are fundamentally triggered by physiological stress ( Figure 1). Physiological stress is an evolutionary conservative coping mechanism involving neuroendocrine responses that put prey in a heightened state of alertness and agility ^21, 22, 49^. If chronic, predation-induced stress can cause prey to change their locomotor biomechanics to enhance escape performance ^50^. Moreover, chronic stress can lead to chronically elevated metabolic rate ^21, 49, 51– 53^. Whether or not prey become chronically stressed can depend on the hunting mode of their predator. Sit-and-wait and sit-and-pursue predators may elicit persistent cues of their presence, triggering a heightened state of alertness and agility ^54^, but a chronic stress response would be energetically wasteful when facing widely roaming predators where encounter frequency is low ^54^.

Elevated metabolism arising from perceived predation risk could change organismal nutrient demand and hence the kinds of resources consumed by prey ^21, 49, 51– 53^, creating a physiological trade-off in nutrient allocation between maintenance (respiration) and both growth and reproduction which alters organismal fitness. Thus, physiological plasticity to increase escape performance entails growth and reproductive costs, which can carry over to influence offspring performance through such things as maternal effects ^22, 48, 50^, but how those costs are borne depends on the capacity of individuals to exhibit adaptive behavior, which is reflected by within-population variation in the degree to which individuals can respond adaptively.

Classic approaches examining trait effects in communities have assumed that traits of an individual are fixed, such that differences in response among phenotypes are continuous and quantitative ^23, 45, 55, 56^. This assumption is made in order to describe responses using continuous frequency distributions of trait values within populations ^45, 55^, but newer research shows that there may be discontinuous differences in trait responses arising from differences among individuals’ morphological, behavioral, or physiological states. This leads to qualitative differences in the way individuals reconcile a trade-off between foraging gains and predator avoidance ^44, 55^.

For example, individuals in low food environments (or in low energetic state or poor body condition) may be motivated to play the trade-off game differently than individuals in high food environments (or in high energetic state or high body condition). Low-energetic-state individuals may accept greater predation risk because starvation risk outweighs predation risk. Alternatively, high-energetic-state individuals may opt to enhance their avoidance of predators ^57, 58^ because they can ride out pulses of risk or are protecting the body condition (asset protection) that they have already built up ^59– 61^. Hence, individuals may be perceived as being shyer or bolder depending on their nutritional or energetic state ^60^. Predators may take advantage of these differences. In spring, predatory barn owls hunt high-energetic-state gerbil prey (prey with large energy stores), giving high energetic return for their foraging effort ^61^. As summer progresses, high-energetic-state gerbils become more vigilant than low-energetic-state gerbils ^61^. While owls still prefer high-energetic-state individuals, they increasingly hunt low-energetic-state individuals, thereby equalizing the hunting pressure on low- and high-energetic-state individuals ^61^.

Shyness and boldness may also be a “personality” trait—a repeatable behavior—of individuals which causes state dependence in predator–prey interactions ^60^ by altering the nature of predator–prey engagement ^62– 66^. Recent research shows that predator and prey personalities essentially amplify outcomes of general predator hunting mode–prey mobility interactions. Personality becomes a key source of trait variation within populations. For example, northern pike predator–stickleback prey interactions involve personality-dependent reciprocal behavioral plasticity ^63^. Pike orient and position themselves to strike moving sticklebacks. Sticklebacks in turn freeze in place (to become cryptic) to fend off an attack. Pike orient longer before attacking when sticklebacks freeze, and the longer stickleback freeze the longer it takes before pike attack ^63^. Hence, individuals that freeze longer (shyer personalities) tend to have higher survivorship, but that outcome is mediated by pike neurophysiology. Individual pike with higher resting metabolic rates (higher energy demands) tend to be more aggressive and strike sooner than individuals with lower metabolic rates ^63^. Predator aggressiveness then favors bolder stickleback individuals that freeze for shorter durations and move to escape. Pike metabolic rate also determines hunting mode and habitat selection: more aggressive individuals tend to engage in active pursuit in the water column, and less aggressive individuals tend to sit-and-wait, hiding in vegetation ^63^. More aggressive individuals also tend to have larger eyes for visual acuity ^63^. Personality also determines contingent outcomes in interactions between black widow spider predators and cricket prey. Bold crickets survive more poorly when facing bold spiders than when facing shy spiders, and vice versa ^64^. Bold crickets seem to escape from spider webs long before shy spiders can subdue them but are quickly captured by bold spiders ^64^. Shy crickets are less likely to move enough to encounter and be caught in webs ^64^. Prey personality can influence outcomes with different predator species as well, as exemplified by interaction between mud crab prey that face active hunting blue crabs and sit-and-wait ambush toad fish ^65^. Bold mud crabs are more likely to succumb to blue crabs because they spend more time outside of refuge habitats, whereas shy mud crabs spend more time in refuge habitats where toad fish tend to lie in wait ^65^. These cases all illustrate how different personality types of predators can select for different prey personality types, preserving phenotypic diversity in both predator and prey populations ^60^.

Phenotypic diversity is also the basis for rapid evolutionary change ^67– 69^, which can lead to another form of state dependence—local adaptation of morphology, behavior, or physiology to environmental context ^44^. A case in point is changes in biomechanical performance in an *Anolis* lizard species. As a clade, arthropod-eating *Anolis* lizard species have adapted to occupy different habitat locations, including the ground, trunks of bushes, and branches. Body and limb morphology can discern which habitat is used. Experimental introductions of a ground-dwelling predatory lizard onto small islands revealed that such differentiation in ecomorphology-habitat association could evolve within-species as well ^67^. The introduced predator selected those individuals of a ground rock-dwelling ambush *Anolis* species that were poorly capable of climbing on trunks and branches ^67^. This triggered plastic changes toward shorter limbs and longer digits of surviving *Anolis* to facilitate active maneuvering on thin branches and catching prey in the higher vegetation canopy. Plasticity became an antecedent to locally adaptive evolutionary change in *Anolis* form and functional role within about 10–15 years, relative to those on control islands ^67^. The interplay between plasticity and adaptive evolution is revealed further in a zooplankton, the water flea *Daphnia*, that has faced different predation regimes ^69^. *Daphnia* produce eggs that often lie dormant in lake sediments. These eggs can be hatched out—“resurrected”—in the lab to ascertain the genotype and phenotype of the parental generation that produced them, which is determined by the environmental conditions experienced by the parental generation ^69^. Generations of eggs, layered upon one another in the sediment, thus store key information about historical changes in environmental conditions within a lake. One historical environmental condition that changes is the presence/absence of fish predators, which can be used to evaluate how *Daphnia* vulnerability traits (for example, body size and shape, length of defensive spines, alertness, and movement in water column) change in response to fish presence (plasticity due to non-consumptive effects) and fish consumption (selection and adaptive evolution) ^69^. Laboratory experiments hatched *Daphnia* individuals from different sediment layers that represented periods before, during, and after fish presence. When the hatched individuals were exposed to fish cues, they expressed different degrees of plasticity and adaptation in vulnerability traits depending on if and when their parental populations were exposed to fish predators within their natal lakes ^69^. Moreover, the degree of plasticity expressed by hatched individuals varied depending on the historical association with fish predators ^69^. This again underscores the need to examine traits in action, including how different evolutionary processes drive the trait changes as environmental context changes in order to enhance predictive understanding of complexity underlying predator–prey dynamics and interactions.

The *Anolis* and *Daphnia* examples, as well as classic studies of Trinidadian guppies evolving different body morphology and coloration to cope with different predation regimes ^70^, reveal that evolutionary processes can be quite rapid. Evolutionary processes can operate contemporaneously with ecological processes, thereby creating eco-evolutionary feedbacks among environmental contexts ^67, 71– 73^. Rapid evolution in response to changing environmental contexts has been documented in a metapopulation of herbivorous stick insect species. In this system, local populations of the stick insect have heritable body coloration patterns that match local patches of their shrub host plants ^38^. One shrub has lance-shaped leaves, and the other has ovoid leaves. Individual stick insects are cryptic to bird predators on lance-leaved shrubs by expressing a dorsal stripe and are cryptic on ovoid-leaved shrubs by expressing a solid green color ^38^. When patches of the different shrubs are in close proximity, gene flow between patches can cause maladaptation in local populations because of misaligned expression of insect body coloration in the shrub ^38^, but more isolated populations exhibit local adaptation. This preserves an eco-evolutionary process that creates a mosaic of stick insect ecotypes across a landscape ^38^. Another recent case involves a lake-dwelling damselfly species. Ancestral forms of the species evolved to coexist with predatory fish ^74^, but this damselfly species has repeatedly invaded fishless lakes containing dragonfly predators. Heritability and selection studies revealed that the damselfly could evolve different predator coping mechanisms within 45 years ^74^. Damselfly larvae in fish lakes evade predators by having low swimming propensity and slow swimming speeds, remaining motionless (hence cryptic) when facing predatory fish that can swim faster ^74^. Damselfly larvae in dragonfly lakes instead swim faster to outrun their predators ^74^.

The rapid pace of human-caused environmental change such as habitat alteration or facilitation of species invasions has increased the likelihood that predator–prey interactions are occurring between species that have not coevolved. Consequently, the traits of native predator and prey species may be poorly adapted for the conditions presented by the new species, whether it is a novel predator or a novel prey ^75, 76^. The new encounters thus could change the relative importance of consumptive and non-consumptive effects that drive the eco-evolutionary game, raising concern about the loss of native predators and prey species and hence the need to manage invasives ^76^. But here too the capacity for plasticity and rapid evolution may enable predator and prey species to cope with these new challenges and hence persist within the newly formed communities ^72, 77, 78^. If this capacity is found to be widespread across predator and prey species, it could change our outlook on the fate of species in a rapidly changing world.

## Conclusions

There is growing appreciation that variety in the structure and functioning of ecological communities and ecosystems can be strongly dependent upon the evolutionary history of the interacting predator and prey species ^67– 74, 79– 82^. The extent to which this reflects variation in the expression of species functional traits that can change via plasticity or rapid evolution in response to the changing ecological conditions created by their interactions ^44, 67, 71^ remains to be seen, and it will likely be difficult to explain context dependency in the absence of taking an adaptive functional trait approach ^40, 44^. This fundamentally requires a new view of predators and prey as organisms, each comprising a suite of traits whose collective function is coordinated—to capture and subdue or evade and defend—as they engage with and adapt to each other in an evolutionary ecological game ^18, 39, 40, 83^.

Thus, understanding the variety inherent in predator–prey interactions requires examining how the game is played in different contexts. This requires taking a new relational experimental approach that observes predator and prey traits in action, requiring analysis of changes in the expression of functional traits within populations of predator and prey species with natural or experimentally imposed changes in ecological contexts ^4, 44^. Such an approach differs from traditional factorial experimental approaches that merely draw prey individuals with different trait magnitudes (for example, body size) from single populations and then experimentally expose them either to predators (or their risk cues) or to cue-free conditions to measure the response of individuals with a given trait. The expanded eco-evolutionary approach discussed here would need to evaluate the potential for local adaptation among predator and prey populations, which includes local adaptation in the nature and strength of phenotypically plastic responses ^44, 67, 74, 75^. This calls for deploying factorial designs using transplant experiments across environmental gradients ^44, 57, 84, 85^. Such experiments would draw individual predators and prey from populations in different environmental conditions and compare the nature and strength of their interactions in transplant as well as native sites to understand patterns of adaptive variation across an environmental landscape as well as the community- and ecosystem-level consequences of their context-dependent interactions.

Ultimately, the adaptive game between predator and prey can be likened to an evolutionary play within an ecological theater ^86^ but which unfolds differently in different theaters (contexts) ^34, 80, 86^.

Hence, the play itself is not scripted but rather is an improvisation that depends on how the players choose to enact the play as well as how their acting changes the look of the theater ^34, 80^. This ultimately depends on the physiological, morphological, and behavioral states of the players ( Figure 1) as well as how quickly the players adapt their traits to each other and the changing theater. Hence, the players and their theaters are in perpetual flux, requiring modern analyses of predator–prey interactions to scale from functional traits to whole ecosystems ^4, 79– 82^ in order to develop a predictive understanding of the variety inherent in predator–prey systems.
